# Supplementary figures and images for: Quantitative trait loci mapping and candidate gene analysis of stoma-related traits in wheat (Triticum aestivum L.) glumes
Source: PeerJ. 2022 Apr 8;10:e13262. doi: 10.7717/peerj.13262 (PMC8997193; doi:10.7717/peerj.13262)

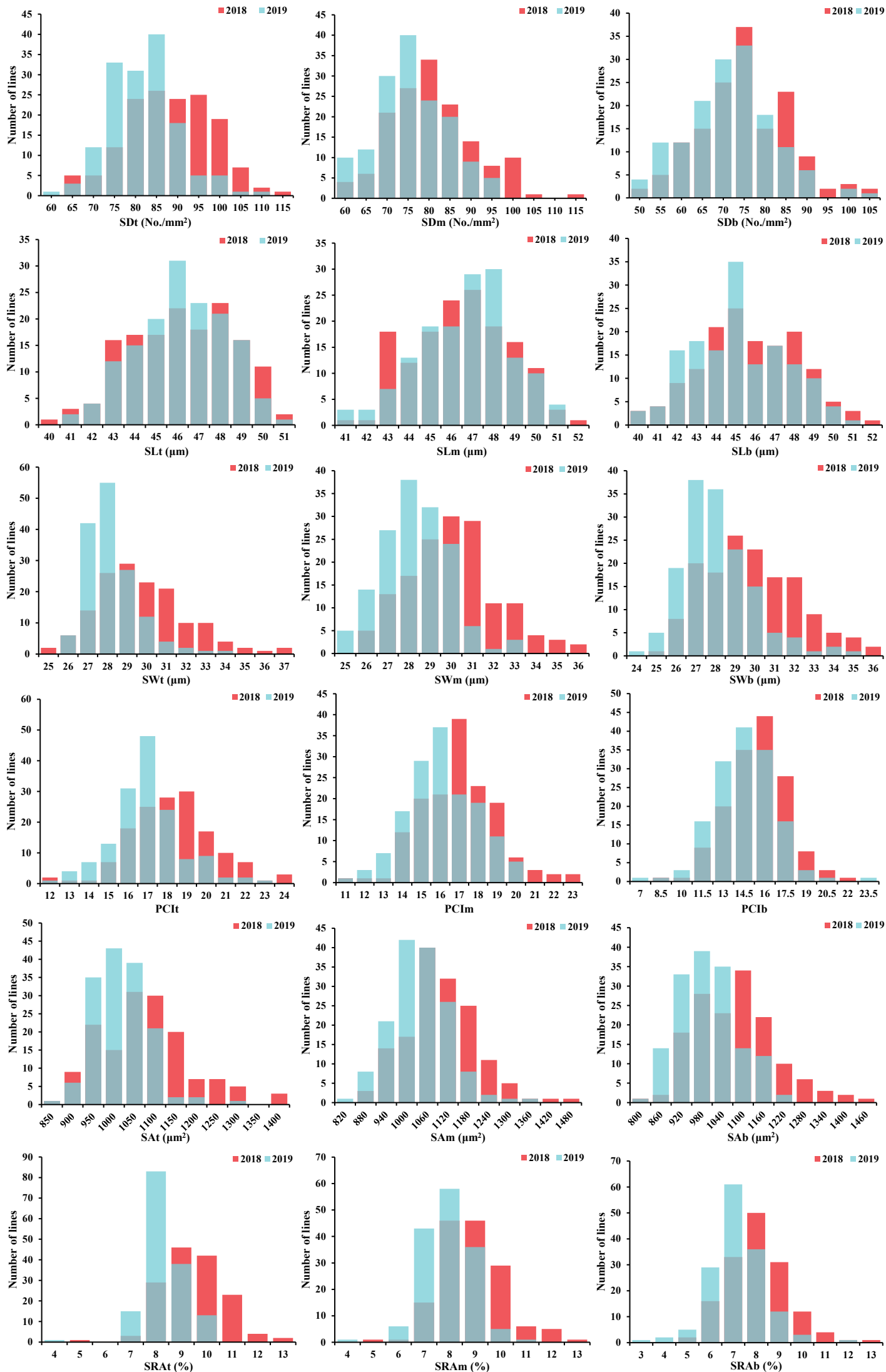

Supplement: Supplemental Information 1 — SD, stomatal density; SL, stomatal length; SW, stomatal width; PCI, potential conductance index; SA, stomatal area; SRA, stomatal relative area; t, top of glumes; m, middle of glumes; b, base of glumes. [file peerj-10-13262-s001.pdf]
