# Supplementary material for: Quantitative trait loci mapping and candidate gene analysis of stoma-related traits in wheat (Triticum aestivum L.) glumes
Source: PeerJ. 2022 Apr 8;10:e13262. doi: 10.7717/peerj.13262 (PMC8997193; doi:10.7717/peerj.13262)
Supplement: Supplemental Information 2 — SD, stomatal density; SL, stomatal length; SW, stomatal width; PCI, Potential conductance index; SA, Stomatal area; SRA, Stomatal relative area; t, top of glume; m, middle of glume; b, base of glume; * and ** indicate significance at p-value <0.05 and p-value <0.01, respectively. The bottom left and top right of the table are the correlation coefficients of 2018 and 2019 respectively. [file peerj-10-13262-s002.docx]

**Table S1 Correlation analysis of stomata-related traits in various parts of glumes**

|  | SDt | SDm | SDb | SLt | SLm | SLb | SWt | SWm | SWb | PCIt | PCIm | PCIb | SAt | SAm | SAb | SRAt | SRAm | SRAb |
| --- | --- | --- | --- | --- | --- | --- | --- | --- | --- | --- | --- | --- | --- | --- | --- | --- | --- | --- |
| SDt | 1 | 0.587** | 0.295** | -0.389** | -0.289** | -0.208* | -0.099 | -0.044 | -0.074 | 0.679** | 0.362** | 0.154 | -0.124 | -0.200* | -0.184* | 0.829** | 0.436** | 0.179* |
| SDm | 0.525** | 1 | 0.495** | -0.384** | -0.380** | -0.226** | -0.077 | -0.027 | 0.034 | 0.282** | 0.699** | 0.334** | -0.088 | -0.245** | -0.109 | 0.421** | 0.797** | 0.405** |
| SDb | 0.254** | 0.500** | 1 | -0.250** | -0.384** | -0.283** | 0.137 | 0.044 | 0.032 | 0.089 | 0.191* | 0.784** | -0.074 | -0.194* | -0.14 | 0.261** | 0.347** | 0.853** |
| SLt | -0.446** | -0.352** | -0.294** | 1 | 0.529** | 0.394** | 0.026 | 0.125 | 0.032 | 0.408** | 0.02 | 0.014 | 0.206* | 0.397** | 0.266** | 0.021 | -0.121 | -0.093 |
| SLm | -0.448** | -0.481** | -0.461** | 0.542** | 1 | 0.459** | 0.105 | 0.231** | 0.112 | 0.139 | 0.391** | -0.072 | 0.224** | 0.743** | 0.364** | -0.034 | 0.105 | -0.165* |
| SLb | -0.385** | -0.397** | -0.366** | 0.475** | 0.621** | 1 | 0.155 | 0.145 | 0.055 | 0.097 | 0.122 | 0.367** | 0.065 | 0.368** | 0.657** | 0.007 | 0.015 | 0.082 |
| SWt | -0.111 | 0.075 | 0.083 | 0.149 | 0.215** | 0.166* | 1 | 0.233** | 0.367** | -0.085 | -0.004 | 0.250** | -0.096 | 0.223** | 0.381** | 0.325** | 0.063 | 0.342** |
| SWm | 0.047 | 0.093 | 0.128 | 0.154 | 0.135 | 0.206* | 0.571** | 1 | 0.285** | 0.052 | 0.139 | 0.144 | 0.02 | 0.821** | 0.306** | 0.096 | 0.478** | 0.204* |
| SWb | 0.004 | 0.024 | 0.021 | 0.155 | 0.174* | 0.253** | 0.489** | 0.584** | 1 | -0.05 | 0.117 | 0.086 | 0.151 | 0.258** | 0.787** | 0.089 | 0.196* | 0.440** |
| PCIt | 0.621** | 0.219** | -0.008 | 0.419** | 0.017 | 0.018 | 0.014 | 0.181* | 0.133 | 1 | 0.386** | 0.152 | 0.051 | 0.118 | 0.021 | 0.835** | 0.342** | 0.095 |
| PCIm | 0.212** | 0.680** | 0.151 | 0.07 | 0.306** | 0.091 | 0.251** | 0.211** | 0.161* | 0.272** | 1 | 0.269** | 0.086 | 0.321** | 0.166* | 0.389** | 0.870** | 0.268** |
| PCIb | -0.042 | 0.191* | 0.712** | 0.069 | 0.008 | 0.386** | 0.205* | 0.284** | 0.212** | 0.005 | 0.213** | 1 | -0.027 | 0.057 | 0.301** | 0.267** | 0.352** | 0.888** |
| SAt | -0.313** | -0.121 | -0.082 | 0.620** | 0.439** | 0.370** | 0.867** | 0.530** | 0.466** | 0.220** | 0.227** | 0.198* | 1 | 0.141 | 0.156 | -0.079 | 0.005 | 0.009 |
| SAm | -0.199* | -0.184* | -0.146 | 0.407** | 0.634** | 0.490** | 0.561** | 0.851** | 0.543** | 0.152 | 0.326** | 0.224** | 0.647** | 1 | 0.421** | 0.047 | 0.386** | 0.043 |
| SAb | -0.195* | -0.185* | -0.168* | 0.356** | 0.443** | 0.691** | 0.453** | 0.538** | 0.873** | 0.106 | 0.163* | 0.352** | 0.539** | 0.651** | 1 | 0.074 | 0.162* | 0.392** |
| SRAt | 0.681** | 0.392** | 0.181* | 0.079 | -0.076 | -0.079 | 0.553** | 0.452** | 0.354** | 0.759** | 0.373** | 0.118 | 0.476** | 0.315** | 0.224** | 1 | 0.431** | 0.289** |
| SRAm | 0.339** | 0.763** | 0.358** | -0.052 | -0.022 | -0.03 | 0.435** | 0.645** | 0.379** | 0.293** | 0.810** | 0.329** | 0.314** | 0.491** | 0.264** | 0.557** | 1 | 0.412** |
| SRAb | 0.106 | 0.317** | 0.754** | -0.018 | -0.112 | 0.135 | 0.383** | 0.481** | 0.597** | 0.081 | 0.245** | 0.851** | 0.294** | 0.312** | 0.513** | 0.329** | 0.499** | 1 |

SD, stomatal density; SL, stomatal length; SW, stomatal width; PCI, Potential conductance index; SA, Stomatal area; SRA, Stomatal relative area; t, top of glume; m, middle of glume; b, base of glume; * and ** indicate significance at p-value <0.05 and p-value <0.01, respectively. The bottom left and top right of the table are the correlation coefficients of 2018 and 2019 respectively.
