# Supplementary material for: Quantitative trait loci mapping and candidate gene analysis of stoma-related traits in wheat (Triticum aestivum L.) glumes
Source: PeerJ. 2022 Apr 8;10:e13262. doi: 10.7717/peerj.13262 (PMC8997193; doi:10.7717/peerj.13262)
Supplement: Supplemental Information 3 [file peerj-10-13262-s003.docx]

Table S2 Genetic information of two QTL hotspots

| **Interval** | **Gene ID** | **Gene annotation** |
| --- | --- | --- |
| AX-109400932~AX-110985652 | *TraesCS6A02G104800* | Transducin family protein / WD-40 repeat family protein |
|  | *TraesCS6A02G104900* | Plant protein 1589 of unknown function |
|  | *TraesCS6A02G105000* | Peroxisomal targeting signal 1 receptor |
|  | *TraesCS6A02G105100* | cytomegalovirus UL139 protein |
|  | *TraesCS6A02G105200* | BTB/POZ/MATH-domain protein |
|  | *TraesCS6A02G105300* | Pol polyprotein |
|  | *TraesCS6A02G105400* | 50S ribosomal protein L3 |
|  | *TraesCS6A02G105500* | BTB/POZ/MATH-domain protein |
|  | *TraesCS6A02G105600* | BTB/POZ/MATH-domain protein |
|  | *TraesCS6A02G105700* | Lectin |
|  | *TraesCS6A02G105800* | Cellulose synthase-like protein |
|  | *TraesCS6A02G105900* | Ectonucleoside triphosphate diphosphohydrolase 1 |
|  | *TraesCS6A02G106000* | Thiosulfate sulfurtransferase GlpE |
|  | *TraesCS6A02G106100* | exocyst complex component sec3A |
|  | *TraesCS6A02G106200* | Cytochrome P450 |
|  | *TraesCS6A02G106300* | Auxin efflux carrier family protein |
|  | *TraesCS6A02G106400* | Stress-associated endoplasmic reticulum protein 2 |
|  | *TraesCS6A02G106500* | Cytochrome P450 family protein |
|  | *TraesCS6A02G106600* | WAT1-related protein |
|  | *TraesCS6A02G106700* | DNA GYRASE B3 |
|  | *TraesCS6A02G106800* | Nodulin-like / Major Facilitator Superfamily protein |
|  | *TraesCS6A02G106900* | Ribonuclease H-like superfamily protein |
|  | *TraesCS6A02G107000* | Pyridoxine/pyridoxamine 5'-phosphate oxidase |
|  | *TraesCS6A02G107100* | Pleiotropic drug resistance ABC transporter |
|  | *TraesCS6A02G107200* | Splicing factor 3B subunit-like protein |
|  | *TraesCS6A02G107300* | Histone acetyltransferase |
|  | *TraesCS6A02G107400* | Ribosomal RNA small subunit methyltransferase H |
|  | *TraesCS6A02G107500* | Serine/threonine-protein kinase |
|  | *TraesCS6A02G107600* | SAGA-associated factor 11 |
|  | *TraesCS6A02G107700* | Photosystem I assembly protein Ycf3 |
|  | *TraesCS6A02G107800* | guanine nucleotide-binding protein subunit gamma |
|  | *TraesCS6A02G107900* | Delta-aminolevulinic acid dehydratase |
|  | *TraesCS6A02G108000* | Leucine-rich repeat protein kinase family protein |
| AX-108972184~AX-108752564 | *TraesCS6A02G113900* | DNA ligase |
|  | *TraesCS6A02G114000* | Receptor protein kinase-like protein |
|  | *TraesCS6A02G114100* | basic helix-loop-helix (bHLH) DNA-binding superfamily protein |
|  | *TraesCS6A02G114200* | Maturase |
|  | *TraesCS6A02G114300* | NAD(P)H-quinone oxidoreductase subunit 2 |
|  | *TraesCS6A02G114400* | 30S ribosomal protein S7 |
|  | *TraesCS6A02G114500* | NADH-ubiquinone oxidoreductase chain 1 |
|  | *TraesCS6A02G114600* | Transport membrane protein |
|  | *TraesCS6A02G114700* | F-box family protein |
|  | *TraesCS6A02G114800* | Cytochrome P450 family protein |
|  | *TraesCS6A02G114900* | ATP-dependent RNA helicase DDX47 |
|  | *TraesCS6A02G115000* | Zinc finger BED domain-containing protein DAYSLEEPER |
|  | *TraesCS6A02G115100* | Purple acid phosphatase |
|  | *TraesCS6A02G115200* | DTW domain containing protein |
|  | *TraesCS6A02G115300* | don-glucosyltransferase 1 |
|  | *TraesCS6A02G115400* | Calcium-dependent lipid-binding (CaLB domain) family |
|  | *TraesCS6A02G115500* | S-adenosyl-L-methionine-dependent methyltransferases superfamily protein |
|  | *TraesCS6A02G115600* | Auxin-responsive family protein |
|  | *TraesCS6A02G115700* | SAUR-like auxin-responsive protein family |
|  | *TraesCS6A02G115800* | DNA/RNA-binding protein KIN17 |
|  | *TraesCS6A02G115900* | Splicing factor 3B subunit 1 |
|  | *TraesCS6A02G116000* | Dolichol kinase |
|  | *TraesCS6A02G116100* | Unknown protein |
|  | *TraesCS6A02G116200* | ATP-dependent RNA helicase |
